# Mechanistic insights into Nrf2-driven pathogenesis and therapeutic targeting in spinal cord injury

**DOI:** 10.3389/fimmu.2025.1574834

**Published:** 2025-07-10

**Authors:** Tao Xie, Jiyu Xu, Xinyu Liu, Yaquan Yu, Yang Lu, Tao Wang, Feng Gao, Hui Yuan

**Affiliations:** ^1^ School of Basic Medical Sciences, Mudanjiang Medical University, Mudanjiang, China; ^2^ School of Clinical Medicine, Jiamusi University, Jiamusi, China; ^3^ School of Medical Imaging, Mudanjiang Medical University, Mudanjiang, China; ^4^ Department of Gastroenterology, Yang Zhou Hong Quan Hospital, Yang Zhou, China; ^5^ Department of Anesthesiology, Mudanjiang Traditional Chinese Medicine Hospital, Mudanjiang, China; ^6^ School of Stomatology, Mudanjiang Medical University, Mudanjiang, China

**Keywords:** spinal cord injury, Nrf2, oxidative stress, inflammation, ferroptosis

## Abstract

Spinal cord injury (SCI) is a traumatic disease of the central nervous system that can result in significant tissue damage and neurological dysfunction. The pathophysiological process of SCI encompasses both primary and secondary injuries, involving various pathological mechanisms such as oxidative stress, inflammation, autophagy, ferroptosis, and mitochondrial dysfunction. Nuclear factor erythroid 2-related factor 2 (Nrf2) is a neuroprotective transcription factor intricately linked to these pathological processes. Upon exposure to external stimuli, Nrf2 undergoes increased nuclear transcription, regulating the expression of various antioxidant genes and directly modulating genes associated with the aforementioned pathological mechanisms to counteract the resultant alterations. Substantial evidence suggests that Nrf2 may be a potential therapeutic target for SCI. Activation of the Nrf2-related signaling pathway effectively inhibits neuronal death following SCI and promotes the recovery of multiple neurological functions. This review provides an overview of recent research on SCI, examines the physiological roles and mechanisms of Nrf2 in SCI, and explores therapeutic strategies targeting this signaling pathway, including non-coding RNAs, natural and synthetic compounds, and other treatments for SCI.

## 1 Introduction

Spinal cord injury is a debilitating condition of the central nervous system caused by either traumatic or non-traumatic factors. It often results in profound motor, sensory, and autonomic dysfunction (1). SCI and its complications impose considerable long-term physical, mental, and economic burdens on patients and their families (2). It is estimated that the global incidence of SCI ranges from 250,000 to 500,000 cases annually. With the rise in extreme sports and traffic accidents, both morbidity and mortality associated with SCI have been increasing, further straining the healthcare system (3–5).

Pathophysiologically, SCI is categorized into two distinct stages: primary injury and secondary injury (6). The former, resulting from the initial trauma to the spinal cord, leads directly to structural damage and nerve tissue loss (7). In contrast, the latter involves a cascade of biological events—including immune cell infiltration, oxidative stress, inflammation, apoptosis, accumulation of excitotoxic neurotransmitters (such as glutamate), disruption of ion homeostasis, and mitochondrial dysfunction—which collectively exacerbate the extent of neuronal damage and promote further neuronal apoptosis (8). Among these mechanisms, immune cell infiltration, inflammatory responses, and oxidative stress play pivotal roles in the progression of SCI. Following the primary injury, vascular damage induces hemorrhage within the spinal cord, which is subsequently followed by the infiltration of peripheral immune cells—such as monocytes, neutrophils, and macrophages—into the spinal tissue (9). These immune cells facilitate the release of inflammatory cytokines, including tumor necrosis factor-α (TNF-α), interleukin-6 (IL-6), and interleukin-1β (IL-1β) (10). The combined effects of immune cell infiltration and cytokine release promote neuroinflammation (11). Although inflammation initially contributes to debris clearance and tissue repair, excessive or prolonged inflammation can result in neuronal apoptosis and further tissue damage (12). Additionally, the destruction of microvessels and the release of hemoglobin from lysed red blood cells lead to the overproduction of reactive oxygen species (ROS), inducing oxidative stress (13). Elevated ROS levels can cause lipid peroxidation, DNA damage, and mitochondrial dysfunction, ultimately triggering iron dysregulation and neuronal apoptosis (14). The interplay between inflammation and oxidative stress further worsens the pathological process, creating a vicious cycle of tissue damage (12). The primary injury occurs immediately upon trauma, resulting in irreversible damage to the affected site. However, the molecular and cellular pathological changes associated with secondary injury are reversible (15). Consequently, the optimal treatment for SCI focuses on inhibiting secondary injury while promoting the recovery of neurological function (16). Current management strategies include surgery, pharmacological interventions, electrical stimulation, cell transplantation, and rehabilitation. However, their effectiveness is limited due to the complex pathophysiology of SCI (17, 18). Therefore, developing effective strategies for treating SCI with secondary injury remains a major challenge.

Nuclear factor erythroid 2-related factor 2 (Nrf2) is a key member of the transcription factor family, ubiquitously expressed across various cell types (19). It reduces cellular damage caused by external stimuli by regulating the transcription of defense genes, playing a crucial role in maintaining intracellular homeostasis (20). Extensive evidence underscores the critical role of Nrf2 in regulating redox homeostasis (21). Under normal conditions, Nrf2 interacts with the E3 ubiquitin ligase complex formed by kelch-like epichlorohydrin-associated protein 1 (Keap1) in the cytoplasm, leading to its ubiquitination and subsequent degradation (22). In response to oxidative stress, Nrf2 is released and translocates to the nucleus, where it binds to the enhancer region of antioxidant response elements (ARE), promoting the expression of antioxidant genes and thereby counteracting oxidative damage (23, 24). Aside from its role in the antioxidant response, Nrf2 is involved in various cellular processes, including inflammation, iron metabolism, DNA repair, autophagy, and mitochondrial membrane integrity (18). Dysregulation of the Nrf2-related signaling pathway can contribute to the onset and progression of multiple pathological conditions, including neurodegenerative disorders, cardiovascular conditions, and cancer (25–27). For example, in Parkinson’s disease, dysregulation of the Nrf2 signaling pathway may exacerbate oxidative stress and neuroinflammation, leading to neuronal damage and cell death (28). Nrf2 activity is intricately regulated through complex transcriptional and post-translational mechanisms, enabling it to coordinate cellular responses and adaptations to diverse pathological stresses and maintain homeostasis (29). In recent years, an increasing number of studies have demonstrated that activation of the Nrf2-related signaling pathway can significantly promote the recovery and improvement of spinal cord nerve function after SCI (30–35).

**Figure 1:**
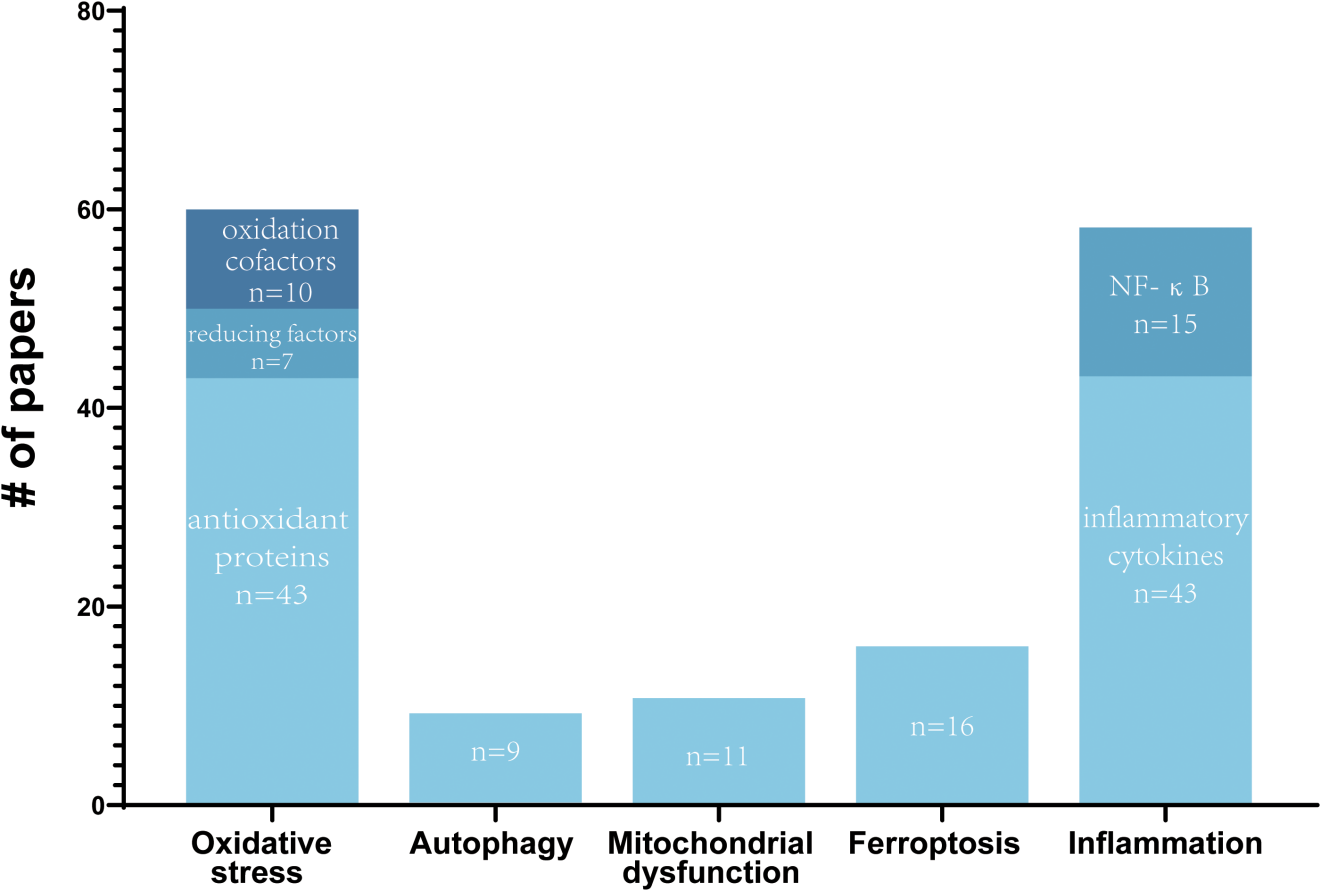
Summary of reported molecular mechanisms of Nrf2 in SCI. The number of papers by reported molecular mechanisms of Nrf2 in SCI are shown. Some papers report multiple molecular mechanisms.

Nrf2 plays a crucial role in secondary injury after SCI (36), numerous studies have demonstrated that Nrf2 enhances the expression of several protective genes such as heme oxygenase-1 (HO-1), superoxide dismutases (SOD), glutathione peroxidase (GPX), catalase (CAT), and NADPH quinone oxidoreductase 1 (NQO1). This action helps mitigate injury and promotes nerve repair after SCI (37–39). Additionally, several non-coding RNAs (ncRNAs), biological macromolecules, and natural or synthetic compounds have been identified as activators of Nrf2 (40–42). These agents regulate the aforementioned pathological changes by activating the Nrf2-related signaling pathway, providing neuroprotective effects. Consequently, Nrf2 may serve as a promising therapeutic target for SCI. This review offers an in-depth analysis of Nrf2’s structure and regulation, while summarizing its mechanisms in SCI (**Figure 1**). Additionally, it explores the potential of ncRNAs, natural or synthetic compounds, biological macromolecules, and other therapeutic strategies to target this signaling pathway for effective SCI treatment.

## 2 The structure of Nrf2 and its upstream regulation in SCI

**Figure 2:**
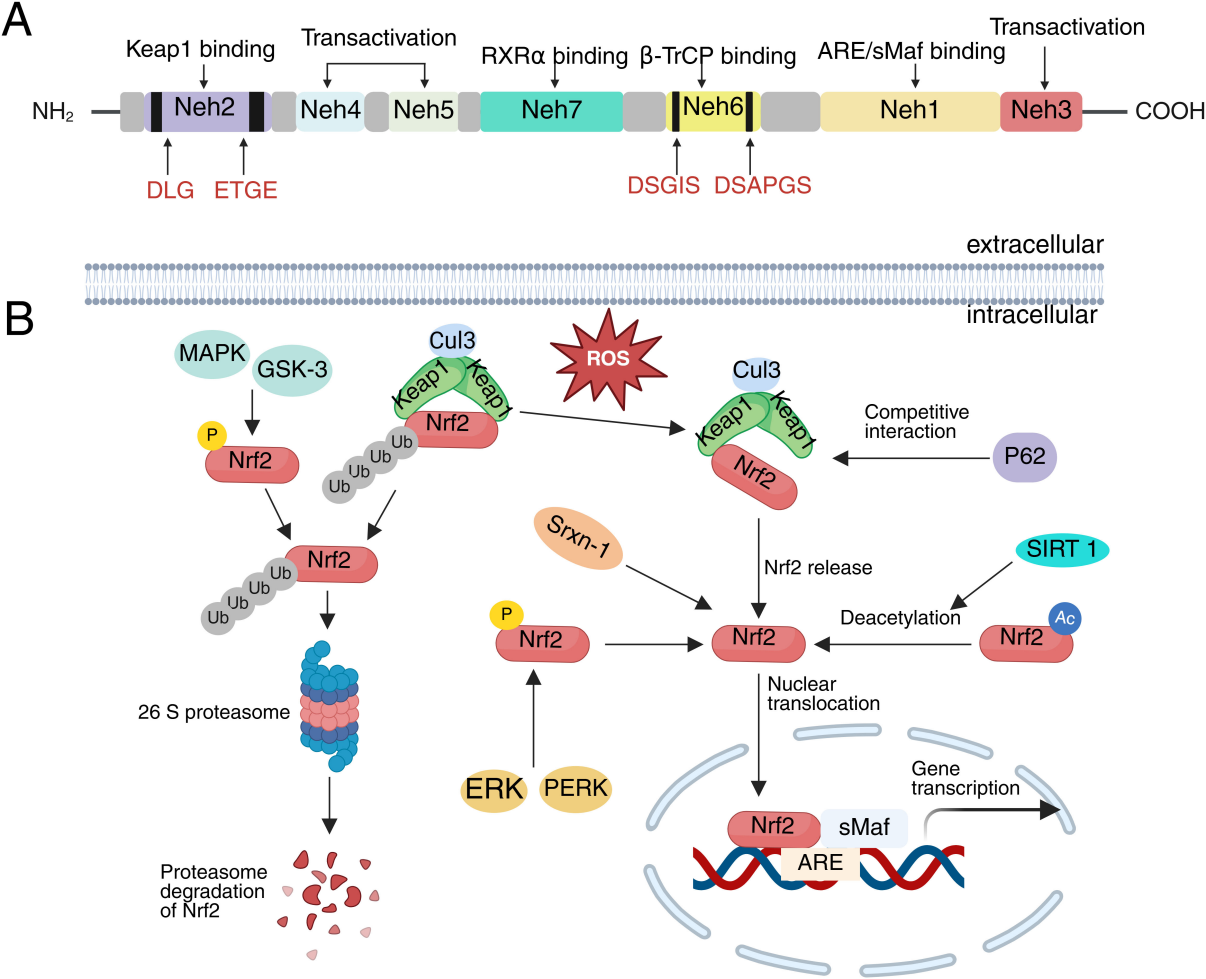
The structure of Nrf2 and its upstream regulation in SCI. **(A)** The domain structure of Nrf2, **(B)** Activation and Degradation of Nrf2.

Nrf2 is a member of the Cap’n’Collar (CNC) family of basic-region leucine zipper (bZIP) transcription factors and is widely expressed across various mammalian cell types (43). The Nrf2 protein, which consists of 605 amino acids, contains seven highly conserved regions known as Nrf2-ECH homology (Neh) domains, labeled Neh1 through Neh7. These domains are crucial for its function in cellular stress responses, particularly in regulating the expression of genes involved in antioxidant defense and detoxification (44). Neh1 includes the CNC-bZIP region, which forms a heterodimer with the leucine zipper motif of the small musculoaponeurotic fibrosarcoma (sMaf) proteins in the nucleus. This heterodimer is responsible for recognizing and binding to the DNA gene sequence on ARE, thereby activating the transcription of target genes and regulating the expression of downstream antioxidant proteins (45). Neh2 interacts with Keap1 via its DLG and ETGE binding units, suppressing Nrf2’s transcriptional activity and promoting its degradation (46). The Neh3 domain, located at the carboxyl terminus of Nrf2, serves as a transactivation domain that interacts with chromodomain helicase DNA-binding protein 6 (CHD6) to sustain Nrf2 transcriptional activity (47). Neh4 and Neh5 domains are independent activation regions that regulate Nrf2 transcription through interactions with the cAMP response element-binding protein (CREB) binding protein (CBP) (48). Neh6 contains DSGIS and DSAPGS motifs that can bind with β-transducin repeat-containing protein (β-TrCP), playing a role in maintaining Nrf2 stability (49). Furthermore, glycogen synthase kinase-3 (GSK-3) enhances Nrf2 ubiquitination and degradation by phosphorylating DSGIS, facilitating the binding of Nrf2 to β-TrCP (50). Neh7 inhibits Nrf2 activity by binding to retinoic X receptor α (RXRα) (51) (**Figure 2A**).

In SCI, the upstream regulatory mechanisms of Nrf2 involve several complexes signaling pathways and molecular interactions. These mechanisms are critical for enabling Nrf2 to exert its cytoprotective effects, as they govern its activation and regulation in response to pathological and physiological changes such as oxidative stress, inflammation, and autophagy induced by SCI (52). The classical activation pathway of Nrf2 is primarily regulated by Keap1, a negative regulator of Nrf2 (45). Under physiological conditions, Keap1 in the cytoplasm binds to the ETGE and DLG motifs of Nrf2 and subsequently interacts with Cul3 via the Neh6 domain of Nrf2, promoting its ubiquitination and rapid degradation by the 26S proteasome (53, 54). Upon stimulation by oxidative stress or electrophiles, cysteine residues within Keap1 undergo oxidation and conformational changes. These alterations lead to an unstable association between Nrf2 and Keap1, thereby inhibiting the ubiquitination and degradation of Nrf2 (55, 56). As a result, Nrf2 is released and translocates into the nucleus, where it heterodimerizes with sMaf. This complex ultimately binds to ARE, inducing transcriptional expression of genes encoding antioxidant enzymes and phase II detoxification enzymes (57). After SCI, Nrf2 is predominantly activated through this classical pathway, exerting a cytoprotective role (58).

Following SCI, in addition to the classical Keap1-Nrf2-ARE pathway for Nrf2 activation, Keap1-independent pathways exist that do not rely on the oxidation of cysteine residues in Keap1 (59). These pathways involve the phosphorylation and deacetylation of Nrf2 through the direct interaction of other proteins with Keap1, leading to the release and activation of Nrf2. Sequestosome 1 (P62/SQSTM1) is a multifunctional protein that binds to microtubule-associated protein light chain 3 (LC3) to guide damaged substrates to autophagosomes for ubiquitination and degradation (60). Notably, P62 and Nrf2 share similar amino acid sequences. When autophagy is impaired following SCI, P62 competes with Nrf2 to bind Keap1, thereby preventing Keap1-mediated degradation of Nrf2 and promoting its nuclear translocation (61, 62). In addition to competitively binding Keap1, P62 can directly promote the degradation of Keap1 through selective autophagy, further activating Nrf2 (63). Sulfiredoxin-1 (Srxn1) is an endogenous antioxidant protein that can directly interact with Nrf2 and facilitate its translocation to the nucleus, thereby increasing its nuclear expression levels (64). Following SCI, this activity of Srxn1 helps to enhance Nrf2 function, improving the cellular response to oxidative stress. In addition, several kinases also phosphorylate Nrf2 and regulate its activity. For instance, protein kinase R (PKR)-like endoplasmic reticulum kinase (PERK) and extracellular regulated kinases (ERK) phosphorylate Nrf2 following SCI and promote its translocation to the nucleus (56, 65–68). Conversely, phosphorylation of Nrf2 by GSK-3β and p38 mitogen-activated protein kinase (MAPK) promotes its degradation (69). Additionally, Sirtuin 1 (SIRT1), a highly conserved NAD+-dependent deacetylase, can directly deacetylate Nrf2, promoting its nuclear translocation and enhancing its ability to bind to DNA, thereby increasing Nrf2 transcriptional activity following SCI (70) (**Figure 2B**). The upstream regulation of Nrf2 in SCI is multifaceted, involving protein-protein interactions, post-translational modifications, and transcriptional control. These regulatory mechanisms contribute to the sustained activation of Nrf2 following SCI. Activated Nrf2 performs multiple protective functions: it upregulates the expression of antioxidant enzymes, thereby mitigating oxidative stress-induced neuronal damage, and suppresses the expression of pro-inflammatory factors, consequently attenuating inflammatory responses (70, 71). Additionally, Nrf2 regulates iron metabolism, reducing the risk of ferroptosis (32). It also promotes a microenvironment conducive to neural tissue repair by maintaining mitochondrial integrity and enhancing autophagy (72). Collectively, these mechanisms underscore the potential of Nrf2 as a key therapeutic target in SCI.

## 3 The role of Nrf2 in spinal cord injury

In the previous section, we discussed the complex structure of Nrf2 and its diverse and tightly regulated mechanisms of action. Many studies have confirmed that the activation of Nrf2-related signaling pathways plays a key role after SCI (73–75). In addition to directly regulating the oxidative stress response, Nrf2 also affects several other pathophysiological processes associated with SCI, such as inflammation, autophagy, iron metabolism, axon regeneration, and mitochondrial function. Given that the genes regulated by Nrf2 are involved in multiple dimensions of SCI pathology, its role in SCI is likely to be complex and multifactorial (75, 76). Therefore, we subsequently summarize the functional contributions of Nrf2 in SCI based on current research findings.

### 3.1 Nrf2 regulates oxidative stress

Primary injury of SCI leads to microvascular rupture, resulting in the destruction and lysis of numerous red blood cells and hemoglobin. Hypoxia and the accumulation of free iron in the injured area stimulate the production of excessive ROS (77). Under normal conditions, ROS are essential for regulating cellular functions such as neuronal activity and cell signaling pathways. However, excessive ROS can lead to lipid peroxidation, DNA damage, inflammation, and cell death, ultimately resulting in severe neurological dysfunction (78).

**Figure 3:**
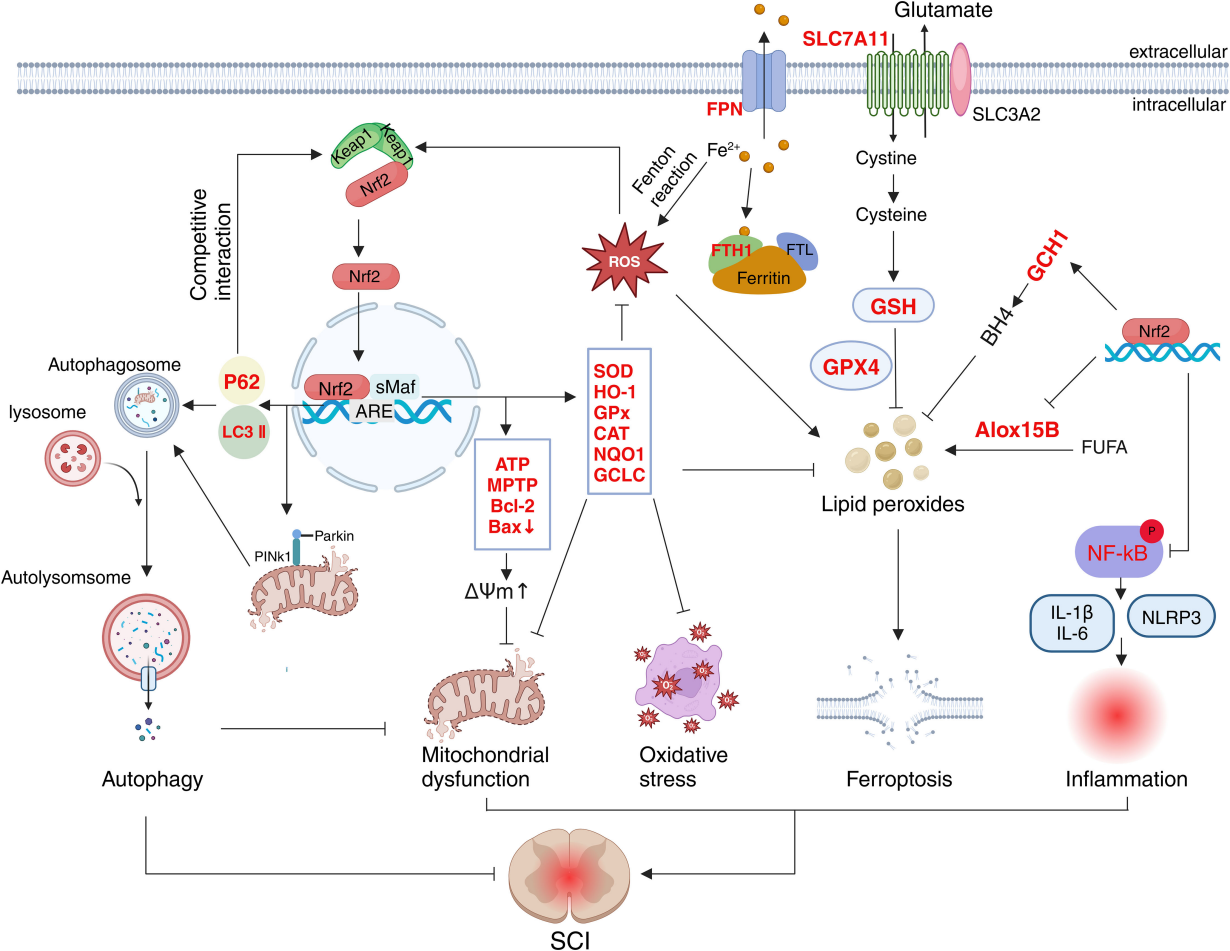
Schematic representation of the multifaceted role of Nrf2 in SCI. The target molecules of Nrf2 (marked in red) are involved in oxidative stress, mitochondrial function, autophagy, inflammation and ferroptosis.

Nrf2 regulates the antioxidant system through multiple pathways, effectively lowering ROS levels following SCI. First, Nrf2 facilitates the breakdown of superoxide and hydrogen peroxide by regulating the activities of SOD, GPX, CAT, and NQO1. For instance, several studies have demonstrated that Nrf2 activation in SCI rat neuronal cells increases the expression of SOD, GPX, CAT, and glutathione (GSH), inhibiting malondialdehyde production and functioning as an antioxidant (79–81). Activation of the Nrf2 pathway also stimulates NQO1 expression. NQO1 catalyzes the reduction of quinones to hydroquinones, facilitating their clearance and preventing ROS generation from quinones via a single-step, double-electron reduction (38, 82). Nrf2 can reduce oxidative stress by boosting the expression of reducing factors. Upon activation, Nrf2 enhances the expression of the glutamate-cysteine ligase catalytic (GCLC) subunit, which plays a key role in regulating GSH synthesis (83). Additionally, Nrf2 helps inhibit oxidative reactions by promoting the regeneration of oxidative cofactors and proteins (42). Furthermore, Nrf2 can directly stimulate the production of antioxidant proteins. For instance, salidroside activates Nrf2, which in turn promotes the expression of thioredoxin 1 (Trx1). This activation inhibits thioredoxin-interacting proteins, thereby exerting an antioxidant effect (84). Experiments have also confirmed that activation of Nrf2 signaling directly targets the ARE elements in the HO-1 and NQO1 genes, regulating their transcriptional activity and inducing the expression of antioxidant proteins (85–87) (**Figure 3**). In summary, Nrf2 mitigates oxidative stress injury after spinal cord injury (SCI) by regulating the expression of various antioxidant enzymes, demonstrating significant potential in reducing lipid peroxidation and preserving cell membrane integrity.

### 3.2 Nrf2 regulates inflammation and immune cell infiltration

In addition to oxidative stress, inflammatory responses mediated by immune cells and inflammatory factors play a critical role in secondary injury following SCI. Local trauma resulting from SCI disrupts the blood-spinal cord barrier (BSCB) and the local microenvironment, leading to the infiltration of monocytes, neutrophils, macrophages, and lymphocytes, as well as the release of inflammatory mediators at the injury site (88). These immune cells promote the secretion of pro-inflammatory cytokines such as TNF-α, IL-1α, IL-1β, and IL-6. Ultimately, the interplay between various immune cells and cytokines results in a severe inflammatory response (15). While inflammation plays a beneficial role in the early stages after SCI, such as debris clearance and tissue repair, excessive immune cell infiltration remains a major contributor to neurological dysfunction (11, 89). After SCI, Nrf2 activation can mitigate the inflammatory response and protect nerve cells by regulating the activity of microglia, monocytes, and macrophages. Studies have demonstrated that activation of Nrf2 can inhibit microglial activation and reduce the expression of inflammatory cytokines, including TNF-α, IL-6, and IL-1β (70, 81, 90). Additionally, Various stimuli following SCI activate inflammasomes, leading to the release of inflammatory factors and subsequent cellular damage. Nrf2 also exerts an inhibitory effect on inflammasome activity. For example, an experiment demonstrated that activation of the Nrf2-related signaling inhibited the production of NLRP3 inflammasomes and increased neuronal survival (91). Notably, Nrf2 also exerts regulatory effects on immune cells such as lymphocytes and neutrophils in other disease models (69, 92, 93). However, there is currently no direct evidence that Nrf2 plays a similar regulatory role in SCI models; therefore, further research is required to confirm this hypothesis.

In addition, Nrf2 participates in neuro-immune interactions by regulating communication between the nervous system and immune cells, which is essential for coordinating inflammation and oxidative stress after SCI. Following SCI, Nrf2 activation in injured neurons and astrocytes promotes the release of neurotrophic factors and antioxidants (94, 95). These neuromodulators act directly on immune cells to inhibit their excessive activation (96). Furthermore, studies have shown that Nrf2 activation in injured neurons reduces oxidative stress and inflammation, thereby decreasing the permeability of the blood-spinal cord barrier (97, 98). Notably, maintaining the integrity of the blood-spinal cord barrier limits the abnormal infiltration of peripheral immune cells and suppresses their pro-inflammatory effects (99). Therefore, it is reasonable to speculate that Nrf2-mediated neuro-immune interaction constitutes a positive feedback loop associated with anti-inflammation and is important for the recovery of neurological function. Additionally, Nrf2 activation in microglia promotes macrophage polarization from the M1 to the M2 phenotype, and M2 macrophages secrete the anti-inflammatory factor interleukin-10 (IL-10), thereby exerting an anti-inflammatory effect during spinal cord repair (86, 100, 101). Conversely, in immune cells, Nrf2 activation can inhibit the NF-κB signaling pathway, thus reducing the release of inflammatory mediators and alleviating inflammatory damage to neurons (102–104) (**Figure 3**). While Nrf2 mechanisms in SCI have been extensively studied, neuro-immune interactions remain underexplored despite their dual role as both drivers of secondary injury and therapeutic targets for neural repair. In-depth investigation of the specific mechanisms of Nrf2 in these interactions may facilitate the identification of new therapeutic targets and strategies, thereby improving the prognosis and quality of life of patients with spinal cord injury.

### 3.3 Nrf2 regulates mitochondrial function

Mitochondrial dysfunction represents another critical pathological process in SCI. Mitochondria, which contain their own unique DNA (mtDNA), are essential for the life activities of nerve cells. They act as the energy powerhouses of the cell and play a key role in regulating fatty acid oxidation, amino acid metabolism, neurogenesis, ROS production, apoptosis, and calcium ion homeostasis (105, 106). Studies have shown that mitochondrial dysfunction is closely associated with the pathological processes after SCI (35, 75, 107). During the acute phase of SCI, substantial changes in mitochondrial morphology and function occur, including mitochondrial swelling, loss of cristae structure, and a more relaxed endoplasmic reticulum. These alterations lead to increased ROS production and reduced ATP synthesis, causing oxidative damage to lipids and DNA, and ultimately triggering neuronal apoptosis (108–111).

Studies have demonstrated that Nrf2 plays a protective role in mitochondrial dysfunction after SCI through various regulatory mechanisms (112, 113). The activation of Nrf2 can induce the expression of multiple antioxidant enzymes and mitigate oxidative stress, thereby safeguarding mitochondria from damage. Experiments have confirmed that Nrf2 activation can promote the production of antioxidant enzymes such as HO-1, SOD2, NQO1, and GSH, thereby protecting mitochondria (100, 114). In nerve cells, Nrf2 can also regulate mitochondrial respiration and NADPH oxidase activity, thereby influencing ROS production (115). In addition to regulating oxidative stress, Nrf2 mitigates mitochondrial damage by regulating apoptosis following SCI. Mitochondrial disruption and membrane potential disorder represent irreversible points in the apoptosis cascade (116). *In vitro* model of spinal cord ischemia/reperfusion injury, researchers found that Nrf2 maintained the stability of mitochondrial membrane potential and prevented mitochondrial swelling and rupture by enhancing mitochondrial permeability transition pore (mPTP) activity and ATP levels (117). Another study has shown that Nrf2 can also maintain membrane potential and mitochondrial membrane stability by increasing anti-apoptotic protein expression and inhibiting pro-apoptotic protein expression, thereby reducing neuronal apoptosis (118). Additionally, Nrf2 activates PINK1/Parkin-mediated mitophagy, promoting the recovery of mitochondrial function (119) (**Figure 3**). As the primary source of cellular energy, mitochondria are essential for most physiological activities (120). Further research is required to determine whether there is a synergistic effect between Nrf2-mediated improvement of mitochondrial dysfunction and other pathophysiological processes, such as oxidative stress and inflammation. Elucidating such interactions may reveal novel therapeutic targets to improve outcomes in SCI management.

### 3.4 Nrf2 regulates ferroptosis

Ferroptosis is also a significant component of the pathogenesis of SCI, further exacerbating neuronal death and dysfunction (121). Ferroptosis is a newly discovered form of iron-dependent programmed cell death, the primary mechanism involves iron metabolism dysregulation and ROS production, leading to PUFA lipid peroxidation, membrane damage, and cell death (122, 123). Glutathione peroxidase 4 (GPX4), a lipid repair enzyme, converts toxic lipid hydroperoxides (L-OOH) to non-toxic lipid alcohols (L-OH) on the cell membrane, thereby mitigating the effects of ferroptosis (124, 125). Additionally, System Xc- (a cystine/glutamate antiporter system) increases cellular uptake of cystine, promoting the synthesis of GSH, which is essential for maintaining GPX4 activity (126–128). Solute carrier family 7 member 11 (SLC7A11/xCT) is a critical subunit of the system Xc- and plays a significant transport role (129). Therefore, GPX4 and its upstream regulators are critical determinants in modulating ferroptosis.

Recent research indicates that ferroptosis is closely related to the pathophysiological process of SCI (121). Following SCI, tissue necrosis and red blood cell destruction at the injury site led to increased local iron ion and ROS levels, resulting in elevated lipid peroxide production and, ultimately, ferroptosis (130). Studies have confirmed that Nrf2 activation promotes the expression of GPX4 and its upstream regulators, thereby reducing lipid peroxide accumulation, inhibiting ferroptosis, promoting neuronal survival, and alleviating SCI symptoms (32, 131). Furthermore, studies revealed that Nrf2 also upregulates GSH and SLC7A11 expression, further inhibiting ferroptosis after SCI (131, 132). In addition, the activation of Nrf2 lowers the levels of free iron ions and boosts the expression of ferroportin (FPN) and ferritin heavy chain 1 (FTH1), which helps to mitigate ferroptosis-related damage following SCI (133). Notably, Nrf2 also inhibited the expression and activity of Alox15B, a lipoxygenase responsible for catalyzing the production of lipid peroxides from polyunsaturated fatty acids in membrane phospholipids, thereby leading to ferroptosis (134). In addition to these mechanisms, Nrf2 promotes the expression of antioxidant enzymes such as SOD, GSH, HO-1, and GPX, thereby reducing oxidative stress and inhibiting ferroptosis (135, 136). Furthermore, Nrf2 activates the GTP cyclohydroxylase 1 (GCH1)/tetrahydrobiopterin (BH4) signaling pathway, reducing oxidative stress and inhibiting ferroptosis. A recent study found that Nrf2 enhances the expression of GCH1, a key enzyme in the synthesis of BH4, which plays a key role in producing superoxide radicals (137–139) (**Figure 3**). Current evidence indicates that Nrf2 activation mitigates ferroptosis and ameliorates SCI through multiple mechanisms. However, systematic investigations into precise mechanisms and potential interactions or synergistic effects among these pathways remain insufficient.

### 3.5 Nrf2 regulates autophagy

Autophagy is an intracellular catabolic process that facilitates the degradation and recycling of damaged proteins, organelles, and other cellular components (140). This process can be broadly categorized into three types: macroautophagy, microautophagy, and chaperone-mediated autophagy, with macroautophagy being the most extensively studied form (141). In response to stimuli such as hypoxia or stress, phagosomes are formed through membrane extensions from the endoplasmic reticulum, Golgi apparatus, and other organelles. These phagosomes encapsulate damaged proteins and organelles to create autophagosomes. Subsequently, autophagosomes fuse with lysosomes to form autolysosomes where the degradation and recycling of damaged proteins and organelles occur (142–144). Autophagy serves as a critical defense mechanism within the body and plays a significant role in the pathophysiological processes of SCI. Most studies suggest that moderate autophagy following SCI helps remove damaged organelles and proteins, maintaining cellular homeostasis and promoting cell survival (145–147). However, other studies indicate that excessive activation or dysregulation of autophagy may have adverse effects, leading to autophagic cell death and exacerbating nerve damage (145, 148). This indicates that investigations of autophagy should extend beyond its cytoprotective functions to include its potential adverse effects. Specifically, it is critical to examine the mechanisms underlying the transition of autophagy from a protective to a harmful role, including abnormal activation of signaling pathways and failures in feedback regulation.

Studies have shown that activation of Nrf2 enhanced the expression of autophagy-related proteins, including P62, LC3, and Beclin-1, thereby promoting autophagy and ultimately exerting a neuroprotective effect (33, 72, 119). Notably, as previously mentioned, P62 can compete with Nrf2 for binding to Keap1, leading to the release and nuclear translocation of Nrf2. Thus, this reciprocal regulation between P62 and Nrf2 forms a positive feedback loop that is significant for cytoprotection. Furthermore, other studies have shown that mitophagy, a specialized form of autophagy, plays a key role in SCI (119, 147). Mitophagy removes damaged or excess mitochondria through autophagy, maintaining mitochondrial number stability and energy metabolism (149) (**Figure 3**). This finding underscores the therapeutic potential of mitophagy in the treatment of spinal cord injuries, as it may protect neurons by modulating mitochondrial quality and energy metabolism. However, the mechanisms underlying the interaction between mitophagy and other cellular processes require further investigation.

### 3.6 Nrf2-mediated protective effects on neuronal populations

Beyond the previously described neuroprotective molecular mechanisms, Nrf2 may also influence specific neuronal populations within the central nervous system (CNS). Neuronal populations refer to collections of neurons within the CNS categorized by characteristics such as origin, function, projection pathways, or molecular features (150). These populations are broadly classified into afferent (sensory) neurons, efferent (motor) neurons, and intrinsic spinal cord neurons. Key efferent populations originating from the brain and brainstem include corticospinal tract (CST), rubrospinal tract (RST), and vestibulospinal tract (VST) neurons (151). Afferent populations encompass sensory neurons, notably those within the dorsal root ganglia (DRG) cells (152). Collectively, these neuronal groups form essential circuits for sensory perception, information integration, and motor execution, thereby mediating the spinal cord’s role in sensorimotor function and adaptation. SCI induces neuronal damage through direct mechanical trauma and secondary injury mechanisms (99). Mechanical injury causes axonal disruption, neuronal cell body damage, and the breakdown of neural networks. Secondary pathological processes—including immune cell infiltration, inflammatory responses, and oxidative stress—exacerbate neuronal death and dysfunction (153). These cascades impair neuronal survival, disrupt synaptic signaling, and perpetuate long-term neurological deficits (13).

As previously mentioned, the activation of Nrf2 can facilitate the recovery of neuronal activity and function following spinal cord injury by modulating immune cells, inflammation, oxidative stress, mitochondrial function, ferroptosis, and autophagy (82, 97, 119). Nrf2 contributes to the survival of neuronal populations and enhances axonal regeneration, partly through mechanisms involving the upregulation of brain-derived neurotrophic factor (BDNF). Consistent with this, research in SCI rat models indicates that Nrf2 activation elevates BDNF expression, correlating with enhanced neuronal plasticity and improved motor function recovery (154, 155). Furthermore, gene network analyses further identify Nrf2 as a critical regulator of axon regeneration-associated genes integral to axonal regeneration, a process fundamental to neurological restoration after traumatic injury (156). Experimental evidence confirms that Nrf2 activation enhances neurological recovery in rodent SCI models by promoting axonal regrowth (34). Mechanistically, Nrf2 upregulates Microtubule-Associated Protein 1B (MAP1B) transcription, a key driver of axon regeneration. MAP1B overexpression and phosphorylation facilitate axonal elongation, while Nrf2-mediated mitochondrial enhancement provides metabolic support for regeneration (157). Concurrently, Nrf2 bolsters neuronal oxidative stress tolerance, improving survival at injury sites (113). Collectively, these mechanisms contribute to functional restoration post-SCI. Although numerous studies underscore the beneficial impact of Nrf2 activation on neuronal populations post-SCI, the specific subpopulations affected and the precise underlying molecular mechanisms warrant further investigation.

## 4 Therapeutic potential of targeting Nrf2 signaling in SCI

Nrf2 is a classical signaling pathway involved in response to oxidative stress, playing a critical role in secondary injury following SCI (18). Aside from its antioxidant functions, Nrf2 also modulates various pathological processes such as inflammation, mitochondrial function, iron metabolism, autophagy, glial scar formation, and axon regeneration after SCI. These actions are essential for the recovery of nerve function post-injury. Consequently, Nrf2 may serve as a potential therapeutic target for SCI. In recent years, numerous studies have demonstrated that various non-coding RNAs, biomolecules, and drugs can activate the Nrf2-related signaling pathway, promoting the recovery of neurological function post-SCI (158, 159).

### 4.1 Non‐coding RNA

Non-coding RNA (ncRNA) refers to a class of RNA molecules that do not encode proteins, including microRNA (miRNA), long non-coding RNA (lncRNA), circular RNA (circRNA), and others (160). These molecules play crucial roles in the regulation of gene expression, chromatin remodeling, RNA processing modifications, and various other biological processes (161). Studies have shown that many miRNAs and lncRNAs regulate the pathophysiological processes of spinal cord injury by activating or inhibiting the Nrf2-related signaling pathway. For instance, previous studies have confirmed that miR-429 is highly expressed in SCI rats, and its inhibition can reduce oxidative stress and inflammation by activating the Nrf2 pathway (159). Additionally, overexpression of lncRNA metastasis-associated lung adenocarcinoma transcript 1 (MALAT1) has been shown to activate Nrf2, promote autophagy, and inhibit neuronal apoptosis (33). Notably, MALAT1 exhibits a contrasting regulatory role in certain tumors (162). However, whether its activation of Nrf2 in SCI could contribute to uncontrolled cell proliferation requires further investigation. According to reports, the expression of lncRNA OIP5-AS1 is decreased in SCI rats, and its overexpression can inhibit ferroptosis and mitochondrial dysfunction, thereby effectively improving SCI (163). Furthermore, other ncRNAs, such as CASC9, MiR-200a, MiR-337, and MiR-101, also contribute to alleviating spinal cord injury by regulating oxidative stress responses, autophagy mechanisms, inflammatory reactions, ferroptosis pathways, and mitochondrial functions (98, 164–166) (**Table 1**).

**Table 1:** Non-coding RNAs affect SCI by regulating Nrf2 signaling pathways.

NcRNA	Overexpression/ inhibition	Function	Reference
MALAT 1	Overexpression	Promote autophagy	(33)
OIP5-AS1	Overexpression	Inhibit ferroptosis and ameliorating	(163)
CASC 9	Overexpression	Inhibit oxidative stress and inflammation	(164)
MiR-200 a	Overexpression	Inhibit oxidative stress	(165)
MiR-337	Inhibition	Inhibit oxidative stress and inflammation	(166)
MiR-429	Inhibition	Inhibit oxidative stress and inflammation	(159)
MiR-101	Overexpression	Repair blood-spinal cord barrier	(98)
MiR-219-5p	Overexpression	Inhibit ferroptosis	(135)
MiR-25	Overexpression	Inhibit oxidative stress	(40)

### 4.2 Natural or synthetic compounds

In recent years, numerous studies have demonstrated that various natural and synthetic compounds derived from plants can exert neuroprotective effects following SCI by activating the Nrf2-related signaling pathway (80, 97, 119, 167, 168). These compounds not only inhibit oxidative stress, inflammation, and ferroptosis, but also reduce glial scar formation, improve mitochondrial function, restore the integrity of the blood-brain barrier, and promote autophagy and axonal regeneration. For example, sulforaphane, an organic sulfide extracted from cruciferous vegetables, exhibits antioxidant and anti-inflammatory effects (169). In SCI rat models, treatment with sulforaphane was shown to mitigate oxidative stress and inflammatory responses by activating Nrf2, thereby playing a crucial neuroprotective role (71). Polydatin is a natural compound with antioxidant effects (170). According to reports, it could alleviate neuronal cell damage induced by mitochondrial dysfunction by activating the Nrf2/ARE pathway after SCI (117). Additionally, Zinc is a trace element in the human body that is essential for neurodevelopment, neurotransmission, and sensory processing (171). Several studies have indicated that zinc enhances Nrf2 expression after SCI, thereby reducing oxidative stress and ferroptosis (32). Ezetimibe is a medication commonly used to treat hypercholesterolemia. Recent research has demonstrated that ezetimibe can upregulate Nrf2 expression post-SCI while inhibiting oxidative stress, inflammatory responses, and glial scar formation (172). Ezetimibe, a drug used to treat hypercholesterolemia, has recently been shown to upregulate Nrf2 expression following spinal cord injury (SCI), thereby reducing oxidative stress, attenuating inflammatory responses, and suppressing glial scar formation [137]. While this finding highlights the potential for repurposing traditional drugs, the specific mechanisms underlying ezetimibe’s protective effects on spinal cord white matter integrity require further investigation. Furthermore, various compounds can activate the Nrf2-related signaling pathway to alleviate SCI (97, 103, 112, 113, 119, 167, 168, 173–185) (**Table 2**).

**Table 2:** Natural/synthetic compounds affect SCI by regulating Nrf2 signaling pathways.

Compound	Function	Reference
Sodium hydrosulphide	Inhibit oxidative stress	(167)
Resveratrol	Inhibit oxidative stress	(173)
Curculigoside	Inhibit oxidative stress	(82)
2-BFI	Inhibit oxidative stress	(80)
Notoginsenoside R1	Inhibit oxidative stress and inflammation	(174)
Biochanin A	Inhibit oxidative stress and inflammation	(72)
Sulforaphane	Inhibit oxidative stress and inflammation	(71)
Albiflorin	Inhibit oxidative stress and inflammation	(175)
Ginsenoside Rg1	Inhibit oxidative stress and inflammation	(79)
Hesperetin	Inhibit oxidative stress and inflammation	(81)
Acacetin	Inhibit oxidative stress and inflammation	(30)
Curcumin	Inhibit oxidative stress and inflammation	(102)
Sinomenine	Inhibit oxidative stress and inflammation	(176)
Rosmarinic acid	Inhibit oxidative stress and inflammation	(104)
Plumbagin	Inhibit oxidative stress and inflammation	(177)
Asiatic acid	Inhibit oxidative stress and inflammation	(178)
Luteolin	Inhibit oxidative stress and inflammation	(179)
Oxyresveratrol	Inhibit oxidative stress and inflammation	(180)
Allicin	Inhibit oxidative stress and inflammation	(181)
Lithium	Inhibit oxidative stress and inflammation	(182)
Perillaldehyde	Inhibit oxidative stress and inflammation	(183)
Isorhamnetin	promote microglia M1 into M2 phenotypen	(168)
Gastrodin	Inhibit inflammation and repair blood-spinal cord barrier	(97)
Polydatin	Reduce oxidative stress and mitochondrial disfunction	(117)
Calycosin	Reduce oxidative stress, inflammation and mitochondrial disfunction	(112)
Maltol	Inhibit oxidative stress and promote mitophagy	(119)
Acteoside	Promote autophagy	(184)
Betulinic acid	Promote autophagy	(147)
Berberine	Inhibit ferroptosis	(136)
Trehalose	Inhibit oxidative stress and ferroptosis	(185)
Zinc	Inhibit oxidative stress and	(32)
Celastro	Reduce Lipid ROS inhibits ferroptosis	(131)
Ezetimibe	Reduce inflammation and glial scar formation	(172)
Forsythoside B	Reduce glial scar formation	(103)
Metformin	Improve Mitochondrial function and axon regeneration	(113)
Morin	Promote axon regeneration	(34)

### 4.3 Biomolecules

Biomolecules are a class of proteins with a wide range of biological activities and significant potential in treating spinal cord injury (186). For example, recombinant human erythropoietin (rhEPO) is a protein hormone generated through genetic recombination technology. Beyond its role in stimulating the production of pre-red blood cells, erythropoietin (EPO) also possesses neuroprotective properties (187). According to reports, rhEPO can increase the expression of NQO1 and glutathione S-transferases (GST) by activating Nrf2, thereby reducing oxidative stress and spinal cord edema (39). Melatonin, a hormone secreted by the human pineal gland, exerts neuroprotective effects on various nervous system diseases (188). Studies have shown that melatonin inhibits the NLRP3 inflammasome by stimulating the Nrf2/ARE pathway, thereby reducing inflammatory responses and mitigating mitochondrial dysfunction (189). SET8 is currently the only lysine methyltransferase identified that can specifically monomethylate the lysine 20 position of histone H4. This modification plays crucial roles in transcription regulation, cell cycle control, and DNA damage repair (190). Overexpression of SET8 can alleviate oxidative stress and reduce glial scar formation by activating the Keap1/Nrf2/ARE pathway (191). Synoviolin 1 (SYVN1) is an E3 ubiquitin ligase involved in various cellular biological processes (192). Experiments have confirmed that SYVN1 overexpression could enhance the activation of the Nrf2/HO-1 signaling pathway and inhibit ferroptosis in a rat model of spinal cord ischemia-reperfusion injury (193). Although this study provides preliminary evidence supporting the potential therapeutic application of SYVN1 in SCI treatment, it does not fully elucidate the regulatory mechanisms through which SYVN1 overexpression modulates ferroptosis-related proteins such as GPX4 and ferroportin. Furthermore, as SYVN1 functions as an E3 ubiquitin ligase, its role in regulating protein homeostasis may be intricately linked to the pathophysiology of SCI. Additional investigations are required to validate these hypotheses and elucidate the underlying mechanisms. Furthermore, we summarized other biomacromolecules that attenuate SCI by activating Nrf2-related signaling pathway (194–197) (**Table 3**).

**Table 3:** Biomolecule affect SCI by regulating Nrf2 signaling pathways.

Biomolecule	Function	Reference
RhEPO	Improve oxidative stress and spinal cord edema	(39)
HSP70	Inhibit oxidative stress	(37)
Teriparatide	Inhibit oxidative stress	(194)
Apoprotein E	Inhibit oxidative stress and inflammation	(195)
Pregnane X receptor	PXR deficiency inhibits oxidative stress, inflammation	(196)
SET8	Inhibit oxidative stress and reduce glial scar formation	(191)
MST1	Improve mitochondrial function	(114)
Melatonin	Reduce inflammation and improve mitochondrial function	(189)
Phosphoglycerate mutase 5	Reduce inflammation and improve mitochondrial function	(35)
FUNDC1	Promote autophagy and improve mitochondrial function	(149)
SYVN1	Inhibit ferroptosis	(193)
GDF15	Inhibit oxidative stress-dependent ferroptosis	(197)

### 4.4 Stem cells, exosomes, and a ketogenic diet

In addition to ncRNAs, compounds, and biological macromolecules, several studies have found that stem cell transplantation, exosomes, and a ketogenic diet can also target Nrf2 and alleviate SCI. For instance, bone mesenchymal stem cells (BMSCs) are multilineage cells capable of self-renewal and differentiation into various cell types, and they are widely found in bone marrow and other tissues (198). Studies have confirmed that the combined treatment of BMSCs and plumbagin could alleviate SCI through the activation of antioxidant, anti-inflammatory, and Nrf2-related signaling pathway (199). In addition, exosomes are vesicles secreted by cells into the extracellular space and are considered potential drug candidates for treating various diseases (200). Studies have found that microglia-derived exosomes (MG-Exos) can induce the expression of downstream antioxidant-related genes such as NQO1, GCLC, and CAT by activating the Nrf2-related signaling pathway, thereby promoting angiogenesis and neurological function recovery after SCI (201). Furthermore, the ketogenic diet is a dietary pattern characterized by high fat, low carbohydrate, and moderate protein intake, which simulates a state of human starvation and uses ketone bodies produced by fat metabolism as an energy source (202). Studies have shown that the ketogenic diet induced Nrf2 activation and reduced oxidative stress and inflammation (203).

## 5 Conclusion

The primary injury in SCI is usually irreversible, whereas the pathological process of the secondary injury can be reversed. Therefore, the treatment strategy for SCI should focus on blocking the progression of the latter and promoting nerve regeneration and functional recovery. Nrf2 can play a neuroprotective role by regulating various pathways. In this review, we provide an in-depth analysis of Nrf2’s structure and regulation, summarize its mechanisms in SCI, and explore the therapeutic potential of targeting this signaling pathway. Activation of the Nrf2-related signaling pathway is beneficial for reducing tissue damage and promoting neurological function recovery after SCI. This involves mechanisms such as antioxidative stress, anti-inflammation, regulation of mitochondrial function, activation of autophagy, and inhibition of ferroptosis. Therefore, we propose that Nrf2 is a valuable therapeutic target. Despite the fundamental understanding of the relationship between the complex pathophysiological mechanisms of SCI and Nrf2-related signaling pathway, some deficiencies and challenges remain that need to be addressed by future studies. First, the pathophysiological changes in SCI are a dynamic process, and different therapeutic strategies may be required to target different stages after injury. Further studies are essential to elucidate the role and regulatory mechanisms of the Nrf2-related signaling pathway at various stages of SCI. Additionally, while numerous natural and synthetic compounds have been identified that target the activation of the Nrf2-related signaling pathway and mitigate SCI in animal or cellular models, current research seldom addresses the long-term effects and potential side effects associated with Nrf2 activation. These factors must be considered for future clinical applications. Furthermore, Collaboration among neuroscientists, molecular biologists, pharmacologists, and clinicians will facilitate the translation of Nrf2-related therapeutics from laboratory settings to clinical practice. Finally, ncRNAs and stem cell therapies have demonstrated promise in treating SCI, however, more comprehensive investigations are required to unravel their complex mechanisms of action and to expand targeted therapeutic options for SCI.
